# Mind the Gap: How Some Viruses Infect Their Hosts

**DOI:** 10.3390/v2112536

**Published:** 2010-11-12

**Authors:** Peter E. Prevelige

**Affiliations:** Department of Microbiology, University of Alabama at Birmingham, Birmingham, AL 35294, USA; E-Mail: prevelig@uab.edu; Tel.: +1-205-975-5327; Fax: +1-205-975-5479

**Keywords:** Cyanophage P-SSP7 phage, DNA injection, cryo-electron microscopy, cryo-electron tomography

## Abstract

Cryo-electron microscopy (Cryo-EM) and cryo-electron tomography (Cryo-ET) provide structural insights into complex biological processes. The podoviridae are dsDNA containing phage with short, non-contractile tails which nevertheless translocate their DNA into the cytoplasm of their host cells. Liu *et al.* [1] used a combination of cryo-EM and cryo-ET to study the structural changes accompanying infection of *P. marinus* by the phage P-SSP7 and thereby provide unique molecular insight into the process by which the DNA transits from phage to host during infection.

There are estimated to be on the order of 10^10^ phage per liter of sea water and roughly 10^24^ phage infections per second [2]. Despite the frequency of phage infection and its ecological importance, relatively little is known about the molecular mechanics of the infection process. Questions such as what provides the driving force for genome exit from the capsid, what signals the conduit to open to allow egress, and how the nucleic acid enters the host cell during infection remain unanswered. However, advances in electron microscopy and image analysis are allowing us to capture a glimpse of this remarkable process. The T7-like podovirus P-SSP7 infects *Prochlorococcus marinus*, the most abundant photosynthetic microorganism. A recent cryo-electron microscopy study by Liu *et al.* [1] provides insight into the molecular details of the P-SSP7 infection process, and given the similarity between P-SSP7 and other podoviruses is likely to provide a paradigm for understanding the process of phage infection.

Cryo-electron microscopy (Cryo-EM), in which biological specimens are rapidly frozen in vitreous ice, provides a way to image biological samples in a frozen hydrated state with a minimum of damage [3]. The images obtained in a transmission electron microscope are two-dimensional projections of three-dimensional objects. In order to obtain a three-dimensional reconstruction it is necessary to view the same object from multiple angles. This can be accomplished by merging images collected from multiple identical objects which have been trapped in the ice in different orientations, a process known incongruously as single particle cryo-EM, or alternatively by tilting the stage to view the same object from multiple angles, a process known as cryo-electron tomography (Cryo-ET). Specimen damage from the electron beam limits the cumulative electron dose and hence the signal to noise. In single particle cryo-EM, the images from tens of thousands of particles obtained at low electron dose might be averaged to obtain a reconstruction which in favorable cases can approach atomic resolution (reviewed in [4]). For symmetrical structures, such as icosahedral viruses, the internal symmetry of the particle means that a single particle image contains multiple views (60 for an icosahedron) of the asymmetric unit in slightly different orientations thereby leveraging the information content of each particle. The tomographic approach, because it views the same object from multiple angles, makes it possible to obtain reconstructions of non-identical objects or events. However, for tomographic reconstructions the number of images of an object that can be obtained is limited by the cumulative beam damage that the object receives with the result that the resolution is typically in the range of nanometers rather than Angstroms.

Liu *et al.* [1] applied both approaches to their study of P-SSP7. Using single molecule reconstruction techniques, 36,000 particle images, and icosahedral averaging, they were able to obtain a 4.6 Å reconstruction of the virus. The capsid displays a triangulation number of seven with the quasiequivalent subunits based on the HK97 fold [5], a fold common in dsDNA containing phage [6]. Single molecule packaging experiments have demonstrated that the DNA inside dsDNA phage capsids is under considerable pressure [7] and the capsids are often reinforced either by chemical crosslinking or the addition of decoration proteins [8]. P-SPP7 does not have either, but apparently stabilizes its capsid through the formation of charge/charge interactions and clamping of one subunit by the N-arm of an adjacent subunit. P-SSP7, like many other dsDNA phage, first forms a procapsid into which the DNA is packaged through a conduit located at one of the 12 icosahedral vertices known as the portal complex [9].

From the perspective of both phage morphogenesis and structure the portal vertex is intriguing. The vertex of the capsid is five-fold symmetric but the portal protein, which replaces the five coat protein subunits at the vertex, is organized as a twelve-fold ring [10]. As a consequence, the portal protein cannot form identical contacts with the surrounding coat protein subunits. This symmetry mismatch is widely conserved leading to the hypothesis that the symmetry mismatch functioned to allow free rotation of the portal protein dodecamer during DNA packaging [11]. However, recent single molecule and protein tethering experiments have demonstrated that the portal protein is unlikely to rotate during DNA packaging [12,13].

To gain insight into the structure of the portal vertex, Liu *et al.* obtained a non-icosahedrally averaged structure of P-SSP7 phage at ∼9 Å resolution (Figure 1). The portal protein subunits have a fold similar to that first observed for phi29 [14] and subsequently seen in the other dsDNA phage [15]. The dodecamer itself displays the typical “grommet” shape and orientation, with the wide end positioned inside the capsid and the narrower stem protruding through the capsid. The stem of the portal dodecamer forms a complex with 12 adaptor protein molecules, which in turn interact with six nozzle protein molecules. The adaptor protein modulates the transition from the 12 fold portal ring to the six fold nozzle complex through a remarkable series of interactions. The nozzle protein has a closed valve which lies proximal to the capsid and DNA can be seen butting up against the closed valve as if ready to translocate.

The wide end or crown region of the portal protein, which lies inside the capsid, contacts 10 surrounding coat protein subunits. The symmetry mismatch is accommodated through variable interactions of the portal protein with the coat protein. The conformation of the 10 coat protein subunits surrounding the portal ring is essentially identical to the equivalent subunits in the other 11 vertices. In contrast, the subunits of the portal dodecamer make four types of non-equivalent contacts with the surrounding coat protein subunits. Given the evidence supporting the incorporation of the portal protein at the early stages of assembly, perhaps through the formation of a scaffolding/portal protein nucleation complex [16], it seems likely that the interaction of the portal with scaffolding proteins allows both sufficient flexibility and bonding energy to recruit coat protein subunits through variable interactions.

A comparison of the structure of phage that had ejected their DNA to those that retained it revealed profound and presumably functional differences at the portal vertex. The tail fibers, which constitute the initial point of interaction with the host cell, change their orientation from one that is inclined upward prior to infection to one that is horizontal post infection. At the same time, there are changes in the portal protein itself and the “adaptor” and “nozzle” proteins. The valve in the nozzle protein is open in the empty capsids, the C-terminal end of the portal protein becomes disordered, and the interaction between the tail fiber and the adaptor protein which lies between the portal and nozzle proteins is broken. Taken together, these structures suggest that alterations in the tail fiber disposition upon host binding are translated to conformational changes in the portal vertex leading to DNA release.

Support for this model was found in tomographic reconstructions of phage caught *in flagrante delicto*. The tail fibers in phage that had attached to the host cell but had not yet injected their DNA were horizontal, similar to those found in empty phage both in solution and those that remained attached to the host cell following injection. The fact that cell bound phage whose tail fibers had triggered but had not yet ejected their DNA were seen suggests that the steps between initial binding and DNA ejection are relatively long lived. A recent tomographic reconstruction of T7-like Podophage e15 in the process of infecting *Salmonella anatum* by the same group revealed a channel like tubular density spanning the outer membrane and periplasmic space [17]. This channel, which is presumably derived in whole or in part from the phage core proteins, most likely functions as a conduit to allow the DNA to pass unharmed through the nuclease rich periplasmic space.

These studies exemplify the exciting developments in cryo-EM and cryo-ET. In the coming years we can look forward to an enhanced understanding, not just of phage or virus infection, but of all manner of biological processes as we continue to develop electron microscope based structural techniques to fill in what has traditionally been a gap between the high resolution structures provided by X-ray crystallography and the cell biology that can be observed by light microscopy.

## Figures and Tables

**Figure 1.**
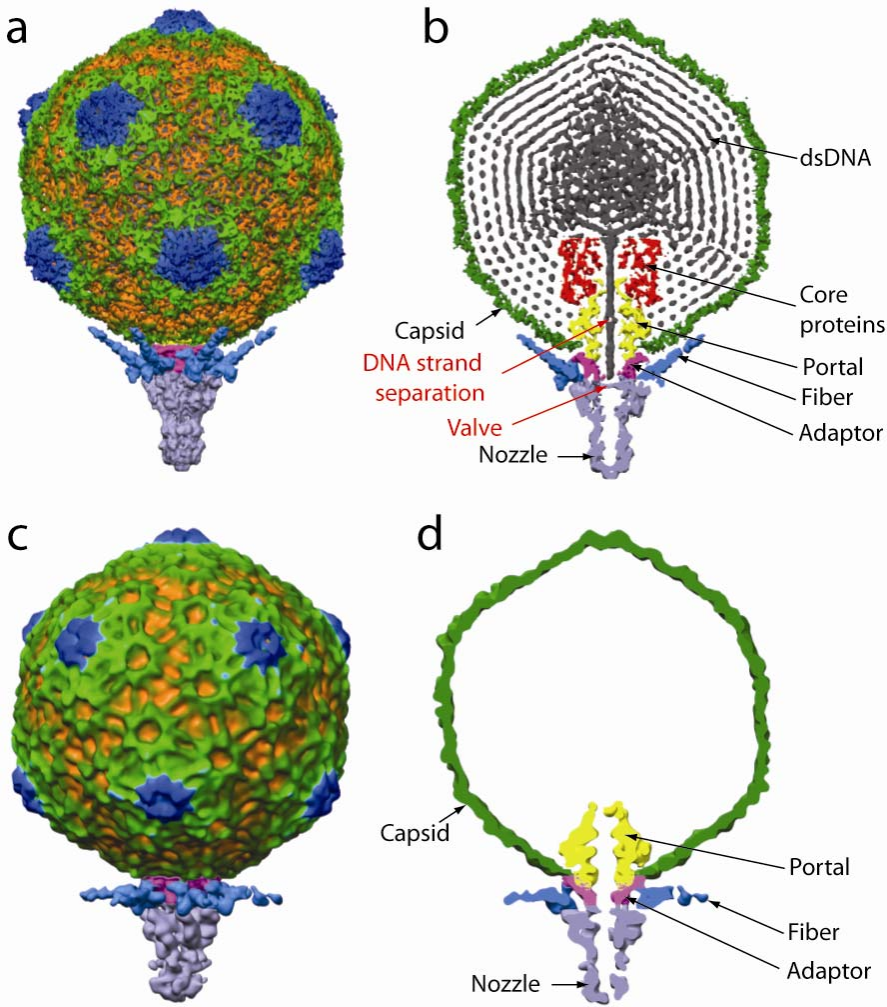
Surface (**a**, **c**) and cutaway (**b**, **d**) views of P-SSP7 full (**a**, **b**) and empty (**c**, **d**) phage. In the full phage the tail fibers are inclined upward whereas in the empty phage they are nearly horizontal to the cell surface. Note also the presence of a closed valve in the full phage and an open valve in the empty phage. Adapted with permission from [1].

## References

[1] Liu X, Zhang Q, Murata K, Baker ML, Sullivan MB, Fu C, Dougherty MT, Schmid MF, Osburne MS, Chisholm SW, Chiu W (2010). Structural changes in a marine podovirus associated with release of its genome into. Prochlorococcus Nat Struct Mol Biol.

[2] Hendrix RW (2003). Bacteriophage genomics. Curr Opin Microbiol.

[3] Lepault J, Booy FP, Dubochet J (1983). Electron microscopy of frozen biological suspensions. J Microsc.

[4] Henderson R (2004). Realizing the potential of electron cryo-microscopy. Q Rev Biophys.

[5] Wikoff WR, Liljas L, Duda RL, Tsuruta H, Hendrix RW, Johnson JE (2000). Topologically linked protein rings in the bacteriophage HK97 capsid. Science.

[6] Morais MC, Choi KH, Koti JS, Chipman PR, Anderson DL, Rossmann MG (2005). Conservation of the capsid structure in tailed dsDNA bacteriophages: the pseudoatomic structure of phi29. Mol Cell.

[7] Smith DE, Tans SJ, Smith SB, Grimes S, Anderson DL, Bustamante C (2001). The bacteriophage phi29 portal motor can package DNA against a large internal force. Nature.

[8] Prevelige PE (2008). Send for reinforcements! Conserved binding of capsid decoration proteins. Structure.

[9] Bazinet C, King J (1985). The DNA translocating vertex of dsDNA bacteriophage. Annu Rev Microbiol.

[10] Valpuesta JM, Carrascosa JL (1994). Structure of viral connectors and their function in bacteriophage assembly and DNA packaging. Q Rev Biophys.

[11] Hendrix RW (1978). Symmetry mismatch and DNA packaging in large bacteriophages. Proc Natl Acad Sci U S A.

[12] Hügel T, Michaelis J, Hetherington CL, Jardine PJ, Grimes S, Walter JM, Falk W, Anderson DL, Bustamante C (2007). Experimental test of Connector Rotation during DNA Packaging into bacteriophage φ29 capsids. PLoS Biol.

[13] Baumann RG, Mullaney J, Black LW (2006). Portal fusion protein constraints on function in DNA packaging of bacteriophage T4. Mol Microbiol.

[14] Simpson AA, Tao Y, Leiman PG, Badasso MO, He Y, Jardine PJ, Olson NH, Morais MC, Grimes S, Anderson DL, Baker TS, Rossmann MG (2000). Structure of the bacteriophage phi29 DNA packaging motor. Nature.

[15] Lebedev AA, Krause MH, Isidro AL, Vagin AA, Orlova EV, Turner J, Dodson EJ, Tavares P, Antson AA (2007). Structural framework for DNA translocation via the viral portal protein. Embo J.

[16] Fu CY, Uetrecht C, Kang S, Morais MC, Heck AJ, Walter MR, Prevelige PE (2010). A docking model based on mass spectrometric and biochemical data describes phage packaging motor incorporation. Mol Cell Proteomics.

[17] Chang JT, Schmid MF, Haase-Pettingell C, Weigele PR, King JA, Chiu W (2010). Visualizing the structural changes of bacteriophage Epsilon15 and its Salmonella host during infection. J Mol Biol.

